# *Alx1* is required for enamel knot formation during incisor morphogenesis

**DOI:** 10.3389/fphys.2026.1939236

**Published:** 2026-09-03

**Authors:** Xi Zheng, Hongbin Lu, Meiyang Chen, Ying Xu, Chensheng Lin, Yi Lv, Yanding Zhang, Xuefeng Hu, Minkui Lin

**Affiliations:** 1 Fujian Key Laboratory of Oral Diseases & Clinical Research Center for Oral Tissue Deficiency Diseases of Fujian Province & Fujian Provincial Engineering Research Center of Oral Biomaterial, School and Hospital of Stomatology, Fujian Medical University, Fuzhou, China; 2 Fujian Key Laboratory of Developmental and Neural Biology, College of Life Sciences, Fujian Normal University, Fuzhou, China

**Keywords:** Alx1, BMP signaling, enamel knot, frontonasal dysplasia, incisor morphogenesis, neural crest-derived mesenchyme, Wnt signaling

## Abstract

**Introduction:**

Mutations in *ALX1* cause frontonasal dysplasia type 3 (FND3), a congenital craniofacial disorder characterized by severe abnormalities of the frontonasal and midfacial structures. However, the role of *ALX1* in tooth development remains unclear.

**Methods:**

We investigated the function of *Alx1* during mouse incisor morphogenesis using a neural crest-specific conditional knockout model. Histological, molecular, and transcriptomic analyses were performed to assess incisor morphogenesis, enamel knot formation, odontogenic gene expression, and signaling pathway activity.

**Results:**

Mutant embryos exhibited complete agenesis of maxillary incisors and impaired development of mandibular incisors. Although tooth initiation was preserved, mutant maxillary incisors failed to progress through the bud-to-cap transition and lacked a discernible enamel knot. Transcriptomic profiling of E13.5 dental mesenchyme revealed alterations in odontogenic gene programs, including genes associated with BMP and Wnt signaling pathways. Consistent with these findings, expression of *Bmp4* and *Msx1* was markedly reduced, accompanied by impaired enamel knot-associated *Shh* expression. In parallel, canonical Wnt signaling activity, as indicated by active β-catenin and LEF1, was attenuated in mutant incisors.

**Discussion:**

These findings identify *Alx1* as an essential regulator of enamel knot formation and incisor morphogenesis, demonstrate a critical role for *Alx1* in maintaining odontogenic signaling during early tooth development, and provide new mechanistic insight into the dental abnormalities associated with *ALX1* deficiency.

## 1 Introduction

Tooth development is a highly orchestrated process that depends on reciprocal interactions between the dental epithelium and neural crest-derived mesenchyme (Balic, 2019; Thesleff, 2003). These epithelial-mesenchymal interactions are coordinated by conserved signaling pathways, including BMP, FGF, SHH, and Wnt signaling, which regulate sequential stages of odontogenesis from initiation to terminal differentiation (Lan et al., 2014; Yu and Klein, 2020; Zhang et al., 2005). A pivotal event during early tooth morphogenesis is the transition from the bud stage to the cap stage, marked by the emergence of the enamel knot, a transient epithelial signaling center that orchestrates tooth growth and patterning through the localized production of morphoregulatory signals (Lan et al., 2014; Thesleff et al., 2001; Vaahtokari et al., 1996). Disruption of enamel knot formation commonly results in developmental arrest and severe tooth malformations, underscoring its central role in odontogenesis (Mogollon et al., 2021).

Extensive genetic studies have demonstrated that transcription factors expressed within the dental mesenchyme are essential for the establishment and maintenance of odontogenic signaling networks. For example, *Msx1* and *Pax9* are indispensable for early tooth development, and deletion of either gene causes developmental arrest at the bud stage (Peters et al., 1998; Satokata and Maas, 1994). Mechanistically, *Msx1* participates in the well-established *Bmp4-Msx1* positive feedback loop in the dental mesenchyme, which acts as a molecular amplifier to propagate odontogenic signals and sustain epithelial-mesenchymal interactions required for enamel knot formation and morphogenetic progression (Chen et al., 1996; Maas and Bei, 1997; Saadi et al., 2013). In parallel, canonical Wnt signaling, mediated by CTNNB1 and its downstream effector LEF1, plays crucial roles in both epithelial and mesenchymal compartments during early odontogenesis (Dassule and McMahon, 1998; Jarvinen et al., 2006; Kratochwil et al., 1996; Lee et al., 2022; O'Connell et al., 2012). Increasing evidence indicates extensive crosstalk between Wnt signaling and mesenchymal transcriptional regulators, including reciprocal interactions between Wnt/β-catenin signaling and MSX1 (Jin et al., 2011; Lee et al., 2022; Medio et al., 2012; Monsoro-Burq et al., 2005; Song et al., 2009).

Despite these advances, the transcriptional mechanisms within neural crest-derived mesenchyme that integrate these signaling pathways to support enamel knot formation and morphogenetic progression remain incompletely understood. Accumulating evidence also indicates that maxillary and mandibular dentitions are regulated by partially distinct developmental programs (Faruangsaeng et al., 2022; Novacescu et al., 2025; Zhang et al., 2005). Genetic perturbations frequently produce unequal effects in upper and lower teeth, suggesting regional differences in signaling requirements and developmental sensitivity (Jia et al., 2013). However, the molecular mechanisms underlying such region-specific regulation remain poorly characterized.

The aristaless-like homeobox gene *ALX1* encodes a transcription factor predominantly expressed in cranial neural crest-derived mesenchyme and is essential for craniofacial development. In humans, biallelic pathogenic variants in *ALX1* cause frontonasal dysplasia type 3 (FND3), which encompasses a spectrum of craniofacial abnormalities of variable severity. Severe *ALX1*-related phenotypes are characterized by extreme microphthalmia, bilateral facial clefting, extensive palatal clefting, and severe abnormalities involving frontonasal and maxillary structures (Pini et al., 2020; Uz et al., 2010). In contrast, a milder form associated with a homozygous splice-site variant has been reported with mouth protrusion accompanied by protruding teeth (Ullah et al., 2017). However, despite these prominent craniofacial and orofacial manifestations, detailed dental phenotyping in individuals with *ALX1*-related FND remains limited, and specific abnormalities of tooth development have not been systematically characterized. Previous studies have therefore primarily focused on the role of *ALX1* in craniofacial morphogenesis and neural crest development, whereas its potential function in odontogenesis has received little attention. Notably, many of the affected craniofacial structures arise from neural crest-derived mesenchyme closely associated with upper jaw and incisor development, suggesting a potential role for *ALX1* in odontogenesis. As a homeobox transcription factor, ALX1 has been implicated in mesenchymal patterning and cell fate specification during embryogenesis (Iyyanar et al., 2026, 2022; Khor and Ettensohn, 2020), raising the possibility that it contributes to the transcriptional networks governing epithelial-mesenchymal communication during tooth development.

In this study, we used a neural crest-specific conditional knockout mouse model to define the role of *Alx1* during incisor morphogenesis and to determine how *Alx1* deficiency affects enamel knot formation and odontogenic signaling. By integrating developmental phenotyping with transcriptomic and molecular analyses, we identify *Alx1* as a previously unrecognized regulator of enamel knot formation and incisor morphogenesis. Our findings establish a developmental role for *Alx1* in incisor morphogenesis and provide a framework for understanding how *ALX1* deficiency may influence odontogenesis.

## 2 Materials and methods

### 2.1 Animals

*Wnt1-Cre* mice were described previously (Danielian et al., 1998). *Alx1^f/f^* mice (C57BL/6J genetic background) were generated by GemPharmatech (Nanjing, China) using CRISPR/Cas9-mediated genome engineering to introduce loxP sites flanking exon 2 of the *Alx1* gene. Neural crest-specific deletion of *Alx1* was achieved by crossing *Alx1^f/f^* mice with *Wnt1-Cre* mice to generate *Wnt1-Cre;Alx1^f/f^* embryos. Mice were maintained under specific pathogen-free (SPF) conditions at 22-24 °C with 40-60% humidity and a 12 h light/dark cycle, with ad libitum access to food and water. Timed matings were established overnight, and the presence of a vaginal plug the following morning was designated as embryonic day 0.5 (E0.5). Embryos were collected at E12.5-E18.5, and pups were collected at P0 for subsequent analysis. Genotyping was performed using genomic DNA extracted from embryonic tails followed by PCR amplification with allele-specific primers for the *Alx1* floxed allele and the Cre transgene. All animal care and experimental procedures were approved by the Institutional Animal Care and Use Committee (IACUC) of Fujian Normal University (Protocol No. IACUC-20230013).

### 2.2 Histological analysis

Embryonic heads were fixed overnight in 4% paraformaldehyde (PFA) at 4 °C. Following fixation, samples were dehydrated through a graded ethanol series, cleared in xylene, and embedded in paraffin. Serial sections were cut at a thickness of 7 μm using a rotary microtome. Hematoxylin/Eosin (H&E) and Azan dichromic staining were performed according to standard protocols. Stained sections were imaged using a bright-field microscope (Olympus BX51, Tokyo, Japan), and representative sections from comparable anatomical levels were selected for analysis.

### 2.3 Whole-mount skeletal staining

P0 WT and *Wnt1-Cre;Alx1^f/f^* pups were euthanized, and the skin, viscera, and subcutaneous tissues were carefully removed. Specimens were fixed in 100% ethanol for 1–2 days and subsequently incubated in acetone for 3 days. Samples were then rinsed three times with distilled water and stained with an Alcian blue-Alizarin red solution for 5 days to visualize cartilage and mineralized bone, respectively. Following staining, specimens were rinsed and immersed in 95% ethanol for 30 min, followed by treatment with 2% KOH to facilitate tissue clearing. Samples were subsequently cleared through a graded glycerol series and stored in glycerol for imaging. Craniofacial skeletal structures were examined using a stereomicroscope (Axio Zoom V16, Carl Zeiss, Germany), and representative dorsal, ventral, and lateral views were photographed.

### 2.4 *In situ* hybridization

Digoxigenin (DIG)-labeled antisense RNA probes for *Alx1*, *Bmp4* and *Shh* were synthesized by *in vitro* transcription using DIG RNA Labeling Mix (11277073910, Roche, Switzerland). Corresponding sense probes were used as negative controls. Paraffin sections were subjected to *in situ* hybridization following a standard DIG-based protocol. Briefly, sections were deparaffinized, treated with proteinase K (10 μg/mL), post-fixed in 4% PFA, acetylated, and prehybridized before overnight hybridization with DIG-labeled probes at 65 °C. Following high-stringency washes and RNase A treatment, sections were incubated overnight at 4 °C with alkaline phosphatase (AP)-conjugated anti-DIG antibody (11093274910, Roche, Switzerland). Hybridization signals were visualized using nitro-blue tetrazolium (NBT) and 5-bromo-4-chloro-3′-indolyphosphate (BCIP) substrates. The color reaction was monitored under a microscope and stopped by washing in TE buffer. Sections were dehydrated, mounted, and imaged using a bright-field microscope (Olympus BX51, Tokyo, Japan) under identical imaging conditions for all experimental groups.

### 2.5 Immunofluorescence

Paraffin sections were deparaffinized, rehydrated, and subjected to heat-mediated antigen retrieval in citrate buffer (10 mM sodium citrate, pH 6.0) at 95-100 °C for 10–15 min, followed by cooling to room temperature. After permeabilization with 0.1% Triton X-100 and blocking with 5% bovine serum albumin (BSA), sections were incubated overnight at 4 °C with primary antibodies against non-phosphorylated (active) β-catenin (1:200, 05-665, Millipore, USA), MSX1 (1:200, AF5045, R&D, USA), and PAX9 (1:200, ab28538, Abcam, UK). Following incubation with Alexa Fluor-conjugated secondary antibodies (Invitrogen, USA), nuclei were counterstained with DAPI (1:500, D1306, Thermo-Fisher, USA). Slides were mounted with antifade mounting medium (H-1000, Vector Laboratories, USA) and imaged using a laser scanning confocal fluorescence microscope (LSM780, Carl Zeiss, Germany) under identical acquisition settings for all samples.

### 2.6 Immunohistochemistry

Immunohistochemistry was performed on paraffin sections following antigen retrieval. Endogenous peroxidase activity was quenched with 3% hydrogen peroxide (H_2_O_2_), and nonspecific binding was blocked using 5% BSA. Sections were incubated overnight at 4 °C with anti-LEF1 antibody (1:200, sc-374522, Santa Cruz, USA), followed by HRP-conjugated secondary antibody (ab6789, Abcam, UK). Immunoreactivity was visualized using a 3, 3′-diaminobenzidine (DAB) substrate kit (ZLI-9019, Zhongshan Jinqiao, China), and sections were counterstained with hematoxylin. Negative controls were processed in parallel by omitting the primary antibody. Images were acquired under identical exposure conditions for all samples.

### 2.7 RNA sequencing and bioinformatic analysis

E13.5 WT and *Wnt1-Cre;Alx1^f/f^* embryos were collected, and the maxillary incisor tooth germs were dissected under a stereomicroscope. The dissected tooth germs were incubated with Dispase II at 37 °C in a humidified incubator with 5% CO_2_ for 15–20 min to facilitate separation of the dental epithelium from the underlying dental mesenchyme. The separation was monitored under a stereomicroscope, and digestion was terminated by transferring the tissues to serum-containing culture medium once the epithelial and mesenchymal compartments were completely separated. The dental mesenchyme was then carefully isolated and collected for RNA extraction. Three independent biological replicates were analyzed for each genotype. Total RNA was extracted using the RNeasy Mini Kit (74106, QIAGEN, Germany) according to the manufacturer’s instructions. RNA quality and integrity were assessed with NanoDrop 2000 spectrophotometer (Thermo-Fisher, USA) and Agilent 2100 Bioanalyzer (Agilent Technologies, USA), and samples with an RNA integrity number (RIN) ≥ 7.0 were used for library construction. RNA sequencing libraries were prepared using the NEBNext Ultra RNA Library Prep Kit for Illumina (New England Biolabs, USA) following poly(A) mRNA enrichment and sequenced on an Illumina NovaSeq 6000 platform to generate paired-end 150-bp reads. Raw reads were quality-checked using FastQC, and adaptor sequences and low-quality reads were removed before alignment to the mouse reference genome (mm10) using HISAT2. Gene-level raw read counts were generated using FeatureCounts. Differentially expressed genes (DEGs) between control and mutant samples were identified using DESeq2 with the raw gene-level count matrix as input. Genes with an adjusted *p*-value < 0.05 and |log_2_(fold change)| ≥ 0.5 were considered significantly differentially expressed. Functional enrichment analysis of DEGs was performed using Gene Ontology (GO) and Kyoto Encyclopedia of Genes and Genomes (KEGG) databases. Enriched biological processes and signaling pathways were identified using standard statistical thresholds (*p* < 0.05). Data visualization, including volcano plots, heatmaps, and enrichment analyses were generated using R packages. Raw sequencing data have been deposited in the Gene Expression Omnibus (GEO) under accession number GSE336212.

### 2.8 Statistical analysis

Differential expression analysis for RNA sequencing data was performed using DESeq2 as described above. Genes with an adjusted *P* value < 0.05 and |log_2_(fold change)| ≥ 0.5 were considered significantly differentially expressed. No additional statistical analyses were performed for histological or imaging data, which were evaluated qualitatively using representative sections from at least three independent embryos per genotype.

## 3 Results

### 3.1 Dynamic spatiotemporal expression pattern of *Alx1* during mouse incisor development

**Figure 1:**
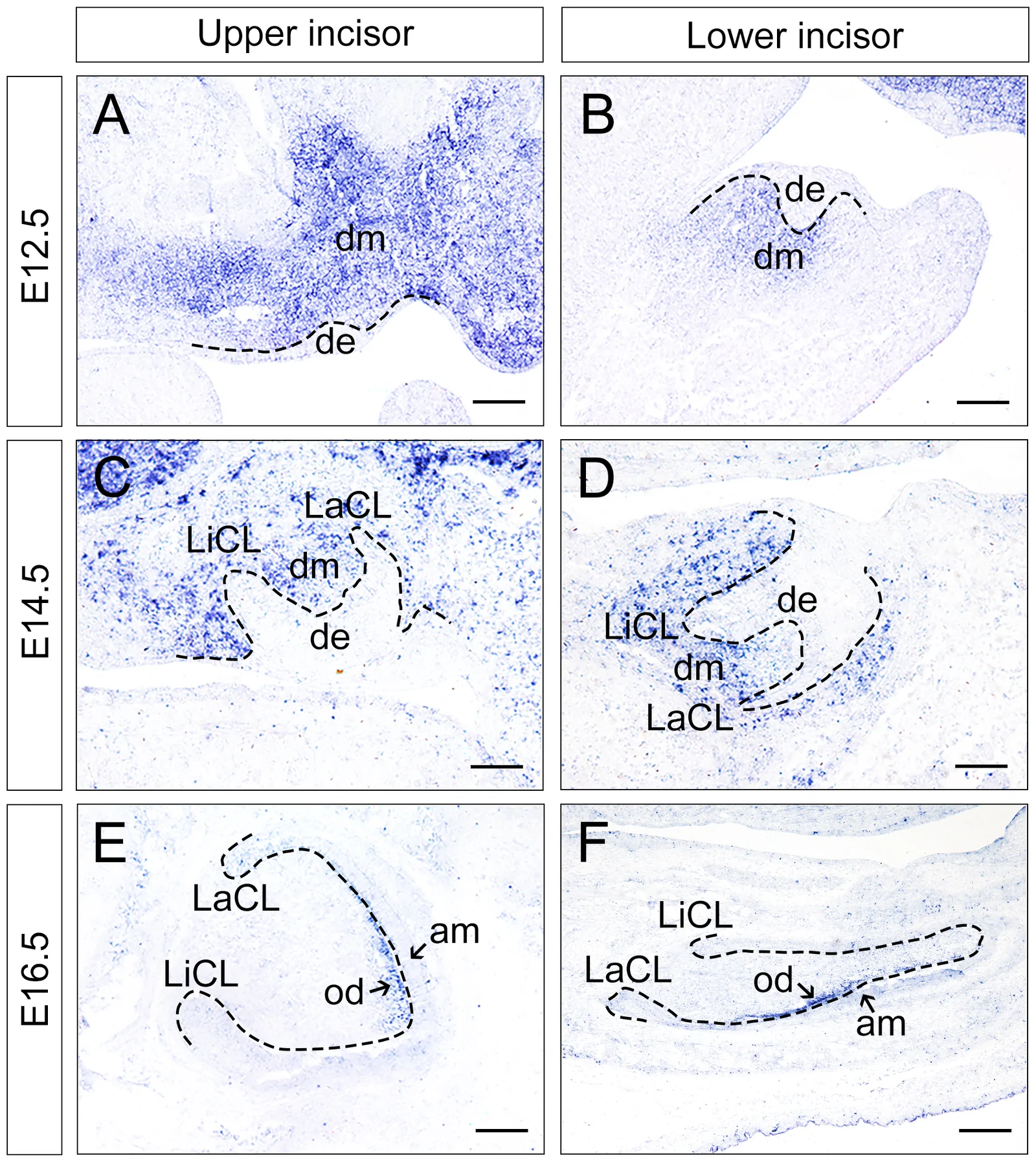
Spatiotemporal expression pattern of *Alx1* during mouse incisor development. Representative *in situ* hybridization images showing *Alx1* expression in developing maxillary and mandibular incisors at E12.5, E14.5, and E16.5. *Alx1* is broadly expressed in the dental mesenchyme at E12.5 and E14.5 **(A–D)** and becomes predominantly restricted to differentiating odontoblasts at E16.5 **(E, F)**. Dashed lines indicate the dental epithelium. am, ameloblasts; de, dental epithelium; dm, dental mesenchyme; LaCL, labial cervical loop; LiCL, lingual cervical loop; od, odontoblasts. Scale bars: 100 μm.

To explore the potential involvement of *Alx1* in tooth development, we first examined its spatiotemporal expression pattern in developing mouse incisors by *in situ* hybridization. At E12.5, *Alx1* transcripts were robustly detected throughout the dental mesenchyme of both maxillary and mandibular incisors, whereas little expression was observed in the dental epithelium (**Figures 1A, B**). This mesenchyme-restricted expression pattern persisted at E14.5, when *Alx1* remained broadly distributed throughout the dental mesenchymal surrounding the invaginating dental epithelium, including regions adjacent to the developing cervical loops (**Figures 1C, D**). At E16.5, however, *Alx1* expression became markedly restricted to differentiating odontoblasts, with minimal expression remaining in other mesenchymal compartments (**Figures 1E, F**). These observations demonstrate that *Alx1* exhibits a dynamic spatiotemporal expression during incisor development, transitioning from broad expression in the early dental mesenchyme to odontoblast-enriched expression at later stages, indicating stage-specific functions during tooth morphogenesis.

### 3.2 Neural crest-specific deletion of *Alx1* results in region-specific defects in incisor morphogenesis

To investigate the developmental function of *Alx1*, we generated neural crest-specific conditional knockout mice (*Wnt1-Cre;Alx1^f/f^*). Efficient deletion of *Alx1* in the dental mesenchyme at E12.5 was confirmed by *in situ* hybridization, which showed a marked loss of *Alx1* expression in mutant embryos (**Supplementary Figure 1**). Mutant mice died shortly after birth owing to cleft palate (data not shown). Notably, examination of the dentition revealed complete absence of the maxillary incisors, indicating an essential requirement for *Alx1* during upper incisor development.

**Figure 2:**
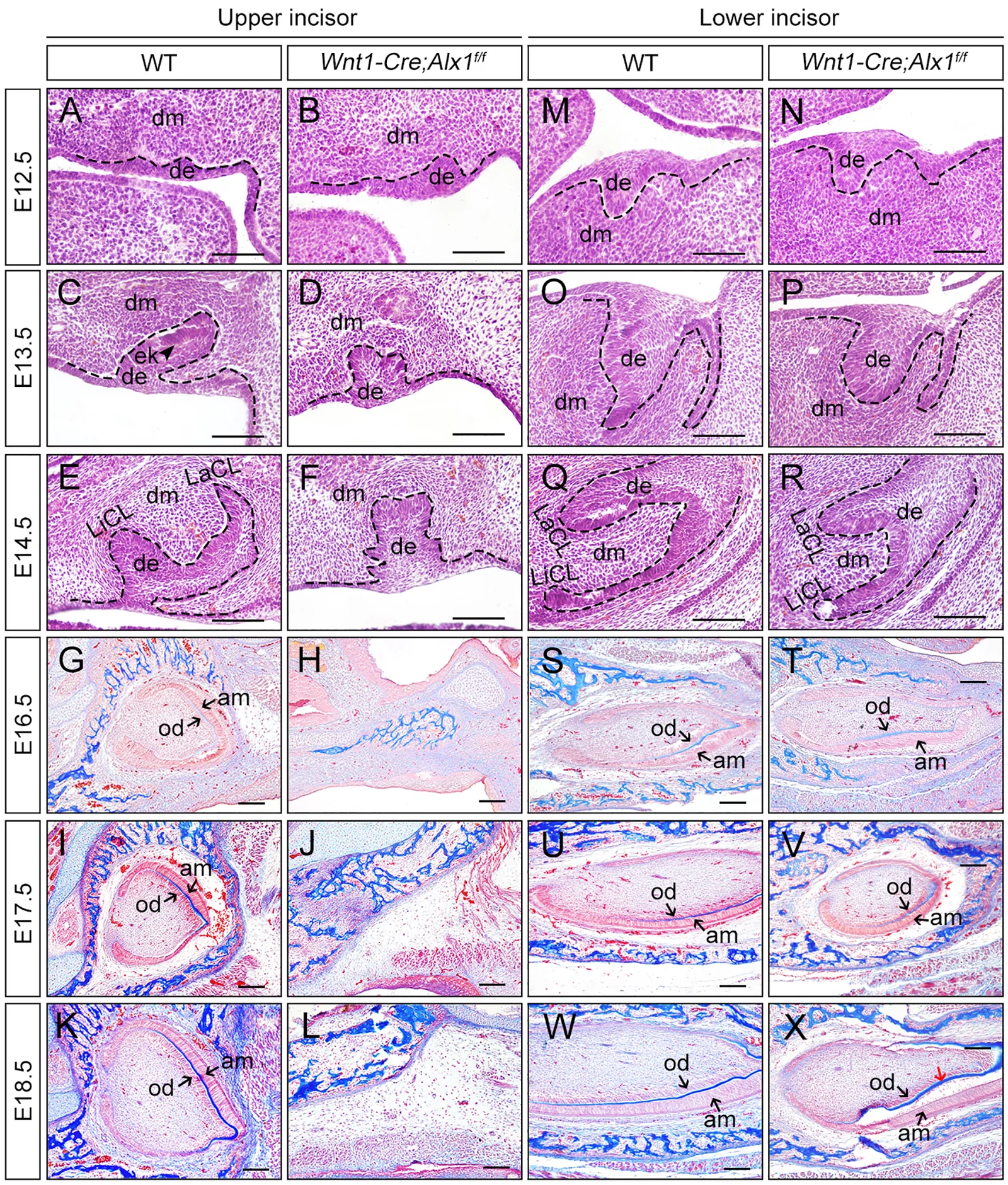
Neural crest-specific deletion of *Alx1* disrupts incisor morphogenesis. Representative hematoxylin and eosin (H&E) and Azan-stained sections of developing maxillary and mandibular incisors from WT and *Wnt1-Cre;Alx1^f/f^* embryos. Maxillary incisors initiate normally at E12.5 **(A, B)** but fail to complete the bud-to-cap transition at E13.5 and E14.5, accompanied by the absence of a discernible enamel knot **(C–F)**. Maxillary incisors are absent at E16.5-E18.5 in mutant embryos **(G–L)**. Mandibular incisors are largely preserved during early development **(M–R)** but exhibit reduced size and increased dentin matrix deposition at later stages [**(S–X)**; red arrows]. Dashed lines indicate the dental epithelium. am, ameloblasts; de, dental epithelium; dm, dental mesenchyme; ek, enamel knot; LaCL, labial cervical loop; LiCL, lingual cervical loop; od, odontoblasts. Scale bars: 100 μm.

Histological analyses demonstrated that tooth initiation was preserved in mutant embryos. At E12.5, both control and mutant maxillary incisors exhibited epithelial thickening and underlying mesenchymal condensation, indicating normal initiation of tooth development (**Figures 2A, B**). At E13.5, control upper incisors displayed pronounced epithelial invagination together with a localized epithelial thickening characteristic of the developing enamel knot (**Figure 2C**). In contrast, mutant upper incisors exhibited reduced epithelial invagination and lacked a discernible epithelial condensation corresponding to the enamel knot (**Figure 2D**). At E14.5, control incisors progressed toward the cap stage, whereas mutant upper incisors remained morphologically similar to an early bud-stage tooth germ and continued to lack enamel knot-like structures (**Figures 2E, F**), indicating failure of the bud-to-cap transition.

In contrast to the severe maxillary phenotype, mandibular incisors developed relatively normally during the early stages. From E12.5 to E14.5, both epithelial invagination and overall tooth morphology were largely preserved, although mutant mandibular incisors appeared slightly smaller than those of control embryos (**Figures 2M–R**). At later developmental stages (E16.5-E18.5), control embryos developed well-organized maxillary incisors, whereas mutant embryos completely lacked maxillary incisor structures (**Figures 2G–L**), demonstrating a fully penetrant developmental arrest. Mutant mandibular incisors, however, were retained but exhibited reduced size and increased dentin matrix deposition, as revealed by Azan staining, without obvious disruption of odontoblast or ameloblast organization (**Figures 2S–X**). To provide additional anatomical context for the dental phenotype, we further examined the craniofacial skeleton of P0 mice by whole-mount skeletal staining. *Wnt1-Cre;Alx1^f/f^* mice exhibited prominent morphological abnormalities in the anterior craniofacial skeleton, particularly in the maxillary/premaxillary region. In addition, the mandibular incisors appeared shorter in mutant mice than in controls, consistent with the reduced size observed histologically (**Supplementary Figure 2**). Together, these findings demonstrate that *Alx1* is dispensable for incisor initiation but is required for normal subsequent morphogenesis, with a particularly critical role in the bud-to-cap transition of maxillary incisors. Importantly, the developmental requirement for *Alx1* is considerably greater in maxillary than in mandibular incisors, revealing a previously unrecognized region-specific role for *Alx1* during odontogenesis.

### 3.3 Transcriptomic profiling identifies coordinated disruption of BMP- and Wnt-associated signaling in *Alx1*-deficient dental mesenchyme

**Figure 3:**
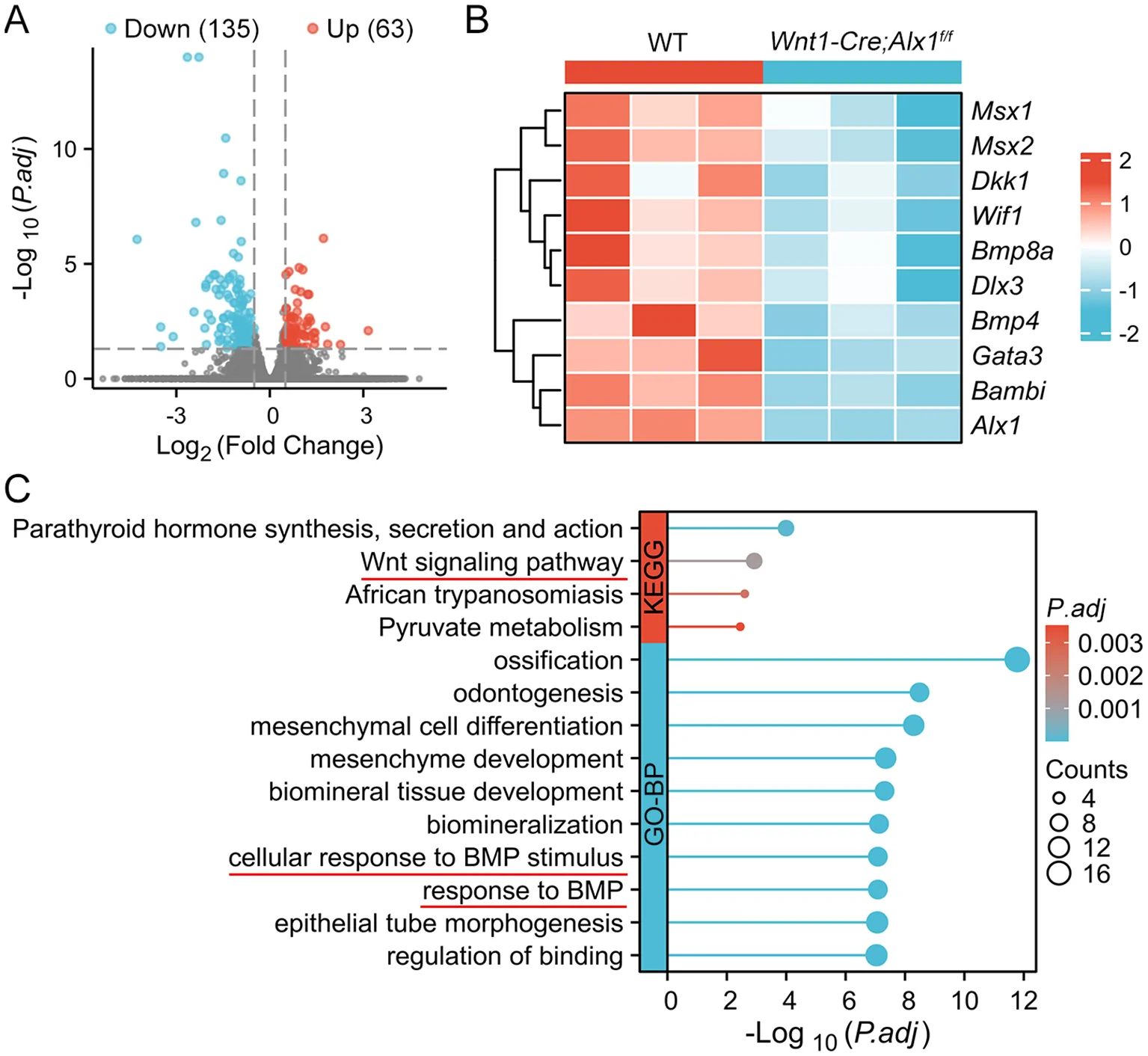
Transcriptomic profiling reveals alterations in odontogenic gene programs in *Alx1*-deficient dental mesenchyme. **(A)** Volcano plot showing differentially expressed genes in dental mesenchyme isolated from E13.5 maxillary incisors of WT and *Wnt1-Cre;Alx1^f/f^* embryos. **(B)** Heatmap showing representative differentially expressed genes associated with odontogenesis and major signaling pathways. **(C)** Gene Ontology (GO) biological process and Kyoto Encyclopedia of Genes and Genomes (KEGG) pathway enrichment analyses of differentially expressed genes, highlighting enrichment of pathways associated with odontogenesis, mesenchymal development, BMP signaling, and Wnt signaling. Dot size represents gene count, and color indicates adjusted *P* value.

To characterize the molecular alterations associated with *Alx1* deficiency, we performed RNA sequencing of dental mesenchyme isolated from E13.5 maxillary incisors. At this stage, WT maxillary incisors are undergoing the bud-to-cap transition, whereas *Wnt1-Cre;Alx1^f/f^* incisors remain at an early bud-like stage. Thus, the transcriptomic comparison captures molecular differences associated with *Alx1* deficiency in the context of impaired developmental progression. Differential expression analysis identified 63 upregulated and 135 downregulated genes in *Wnt1-Cre;Alx1^f/f^* embryos compared with WT controls (**Figure 3A**). Heatmap analysis revealed coordinated downregulation of multiple odontogenic regulators, including *Bmp4*, *Msx1*, and *Msx2*, together with Wnt- associated modulators such as *Wif1* and *Dkk1* (**Figure 3B**). Functional enrichment analyses further demonstrated significant enrichment of biological processes related to BMP signaling, epithelial tube morphogenesis, and mesenchymal development in Gene Ontology (GO) analysis, whereas Kyoto Encyclopedia of Genes and Genomes (KEGG) analysis identified the Wnt signaling pathway as one of the most significantly enriched pathways (**Figure 3C**). Together, these transcriptomic analyses show that *Alx1* deficiency is associated with coordinated alterations in multiple odontogenic gene programs, including BMP- and Wnt-related pathways, at a stage when mutant maxillary incisors have already begun to diverge morphologically from WT controls.

### 3.4 *Alx1* deficiency impairs enamel knot-associated expression of *Shh* and *Bmp4*

Given the absence of a discernible enamel knot in *Alx1*-deficient maxillary incisors and the transcriptomic evidence implicating disruption of BMP- and Wnt-associated pathways, we next examined the spatial expression of two key enamel knot-associated signaling molecules, *Shh* and *Bmp4* (Jernvall et al., 1998; Nunes et al., 2007).

**Figure 4:**
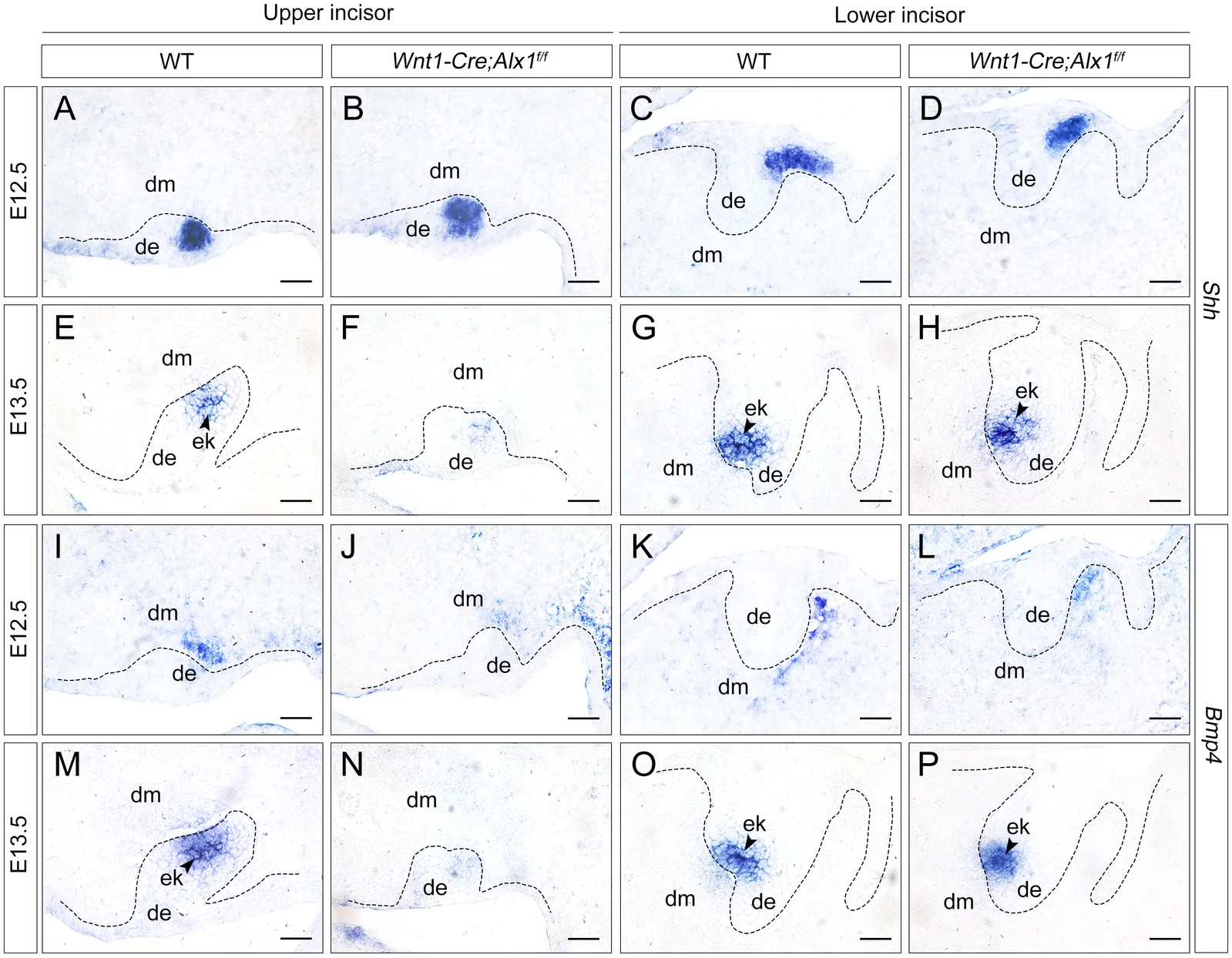
*Alx1* deficiency impairs enamel knot-associated *Shh* and *Bmp4* expression. Representative *in situ* hybridization images showing *Shh* and *Bmp4* expression in developing maxillary and mandibular incisors of WT and *Wnt1-Cre;Alx1^f/f^* embryos. **(A–H)** *Shh* expression at E12.5 and E13.5. A restricted *Shh*-positive domain is established in the enamel knot of control maxillary incisors at E13.5 **(E)** but is absent in mutant embryos **(F)**. **(I–P)** *Bmp4* expression at E12.5 and E13.5. Epithelial localization of *Bmp4* in the enamel knot is observed in control maxillary incisors **(M)** but is lost following *Alx1* deletion **(N)**. Dashed lines indicate the dental epithelium. de, dental epithelium; dm, dental mesenchyme; ek, enamel knot. Scale bars: 100 μm.

At E12.5, *Shh* expression was detected in the dental epithelium of both maxillary and mandibular incisors and appeared comparable between control and mutant embryos (**Figures 4A–D**). At E13.5, *Shh* expression in control maxillary incisors became highly restricted to the enamel knot region within the dental epithelium (**Figure 4E**), consistent with previous studies identifying Shh as a characteristic marker of enamel knot signaling activity (Vaahtokari et al., 1996). In contrast, mutant maxillary incisors exhibited a marked reduction of *Shh* expression and failed to establish a distinct enamel knot-associated signaling domain (**Figure 4F**). In mandibular incisors, the spatial distribution of *Shh* remained largely comparable between control and mutant embryos (**Figures 4G, H**).

We next examined *Bmp4*, a critical regulator of epithelial-mesenchymal signaling during enamel knot formation (Jernvall et al., 1998). At E12.5, *Bmp4* was predominantly expressed in the dental mesenchyme of both maxillary and mandibular incisors. Although expression in mandibular incisors remained largely unchanged, *Bmp4* expression was moderately reduced in the maxillary dental mesenchyme of mutant embryos (**Figures 4I–L**). At E13.5, *Bmp4* expression in control maxillary incisors became strongly localized to the enamel knot (**Figure 4M**), whereas this epithelial expression was almost completely absent in mutant embryos (**Figure 4N**). In mandibular incisors, *Bmp4* expression was largely preserved, with only a modest reduction in signal intensity (**Figures 4O, P**). Collectively, these findings demonstrate that loss of *Alx1* disrupts the establishment of enamel knot-associated signaling, characterized by impaired *Shh* and *Bmp4* expression, particularly during maxillary incisor development.

### 3.5 *Alx1* deficiency selectively reduces MSX1 expression while preserving PAX9 expression

**Figure 5:**
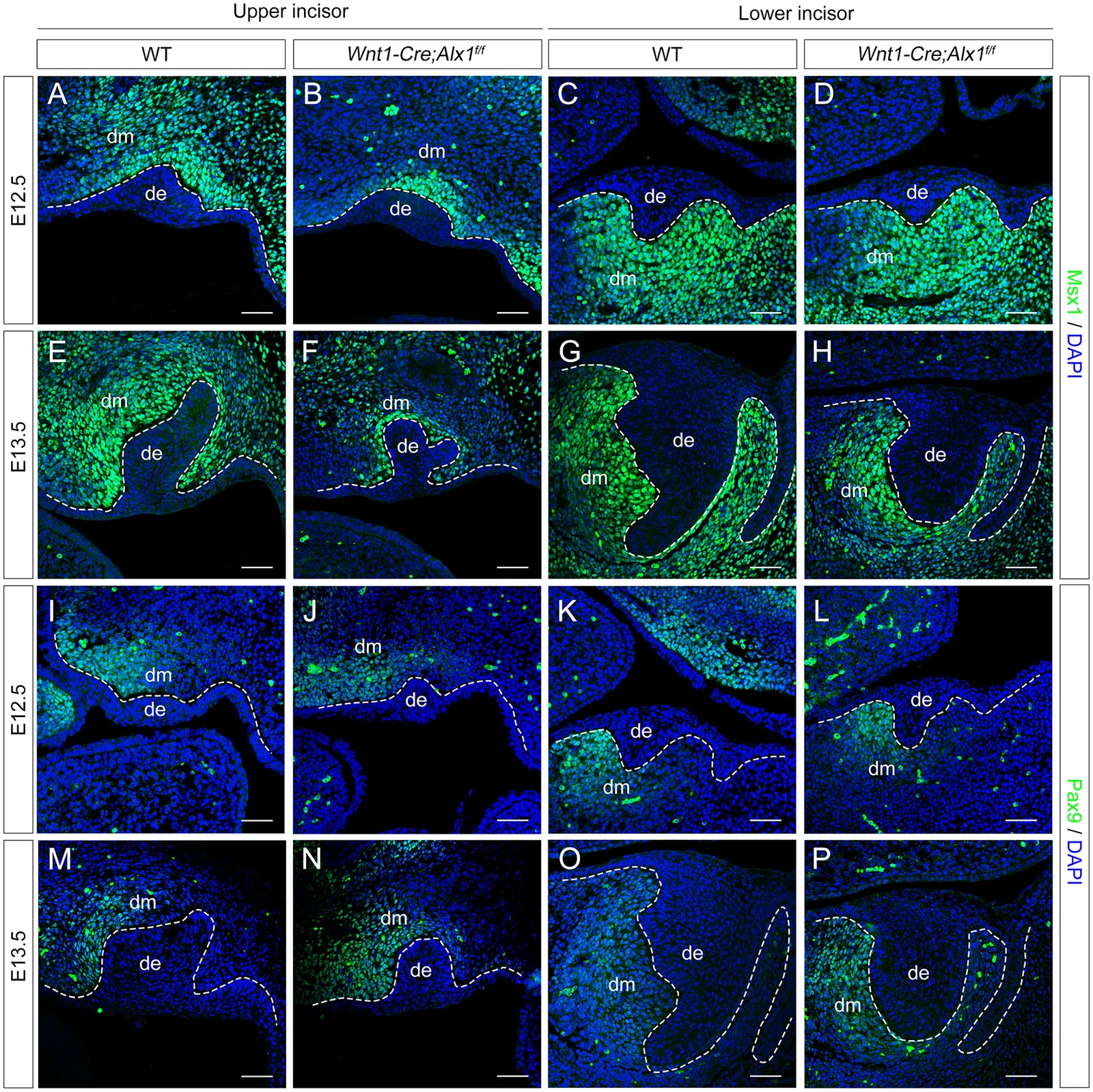
Reduced MSX1 expression but preserved PAX9 expression in *Alx1*-deficient incisors. Representative immunofluorescence images showing MSX1 and PAX9 expression in developing maxillary and mandibular incisors of WT and *Wnt1-Cre;Alx1^f/f^* embryos at E12.5 and E13.5. **(A–H)** MSX1 expression is reduced in mutant maxillary incisors at both developmental stages, whereas mandibular incisors retain a similar spatial distribution with decreased signal intensity. **(I–P)** PAX9 expression remains largely comparable between WT and mutant embryos in both maxillary and mandibular incisors. Dashed lines indicate the dental epithelium. de, dental epithelium; dm, dental mesenchyme. Scale bars: 100 μm.

Because MSX1 and PAX9 are key mesenchymal transcription factors required for early tooth development, we next examined whether *Alx1* deficiency broadly disrupts the odontogenic transcriptional network or selectively affects specific transcriptional regulators. At E12.5, MSX1 was robustly expressed throughout the dental mesenchyme of both maxillary and mandibular incisors in control embryos. In mutant embryos, MSX1 expression was moderately reduced in the maxillary dental mesenchyme, whereas expression in mandibular incisors remained largely comparable to controls (**Figures 5A–D**). At E13.5, MSX1 expression was markedly decreased in the maxillary dental mesenchyme of mutant embryos (**Figures 5E, F**). A modest reduction was also observed in mandibular incisors, although the overall spatial distribution remained largely preserved (**Figures 5G, H**).

In contrast, the spatial distribution and overall PAX9 immunostaining pattern appeared largely preserved following *Alx1* deletion. Comparable PAX9 expression patterns were observed in control and mutant embryos at E12.5 and E13.5 in both maxillary and mandibular incisors, with only subtle differences in signal intensity (**Figures 5I–P**). Together, these findings indicate that *Alx1* deficiency does not broadly impair the odontogenic transcription factor network but selectively reduces MSX1 expression while preserving PAX9 expression, suggesting that *Alx1* preferentially influences specific mesenchymal regulatory programs during early incisor development.

### 3.6 *Alx1* deficiency attenuates canonical Wnt signaling during early incisor morphogenesis

**Figure 6:**
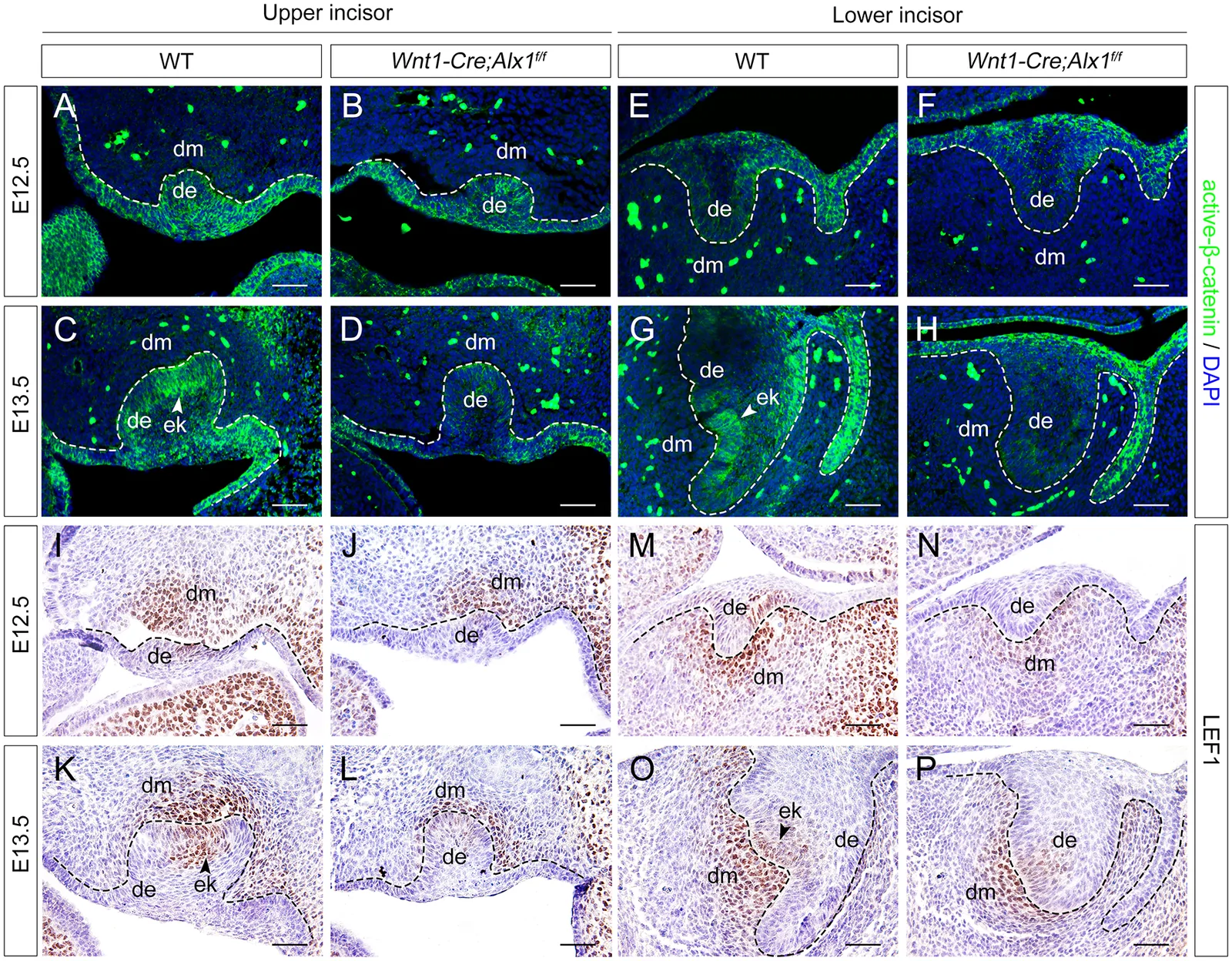
Attenuated canonical Wnt signaling in *Alx1*-deficient incisors. Representative immunofluorescence and immunohistochemical images showing canonical Wnt signaling activity in developing maxillary and mandibular incisors of WT and *Wnt1-Cre;Alx1^f/f^* embryos at E12.5 and E13.5. **(A–H)** Immunofluorescence staining for non-phosphorylated (active) β-catenin, a marker of canonical Wnt/β-catenin signaling activity. Enrichment of active β-catenin in the enamel knot region of control maxillary incisors at E13.5 **(C)** is absent following *Alx1* deletion **(D)**. **(I–P)** Immunohistochemical staining for LEF1. Control maxillary incisors exhibit prominent LEF1 expression within the enamel knot region at E13.5 **(K)**, whereas this localized enrichment is not observed in mutant embryos **(L)**. Dashed lines indicate the dental epithelium. de, dental epithelium; dm, dental mesenchyme; ek, enamel knot. Scale bars: 100 μm.

Given that canonical Wnt/β-catenin signaling has been shown to both regulate and be regulated by MSX1, forming a bidirectional regulatory relationship (Jin et al., 2011; Lee et al., 2022; Medio et al., 2012; Monsoro-Burq et al., 2005; Song et al., 2009), we next examined its activity in *Alx1*-deficient incisors using an antibody recognizing the non-phosphorylated (active) form of β-catenin, together with LEF1 immunostaining. At E12.5, active β-catenin was detected in both the dental epithelium and mesenchyme of control maxillary incisors, with stronger immunoreactivity in the epithelium (**Figure 6A**). In mutant embryos, active β-catenin expression was reduced in both tissue compartments while maintaining a similar spatial distribution (**Figure 6B**). At E13.5, active β-catenin became strongly enriched within the enamel knot region of control maxillary incisors (**Figure 6C**). In contrast, mutant maxillary incisors exhibited markedly reduced active β-catenin expression and failed to establish the characteristic epithelial enrichment associated with the enamel knot (**Figure 6D**). In mandibular incisors, active β-catenin expression was largely preserved but showed a moderate reduction in signal intensity at both developmental stages (**Figures 6E–H**).

Consistent with these observations, LEF1 immunostaining demonstrated robust expression in both epithelial and mesenchymal compartments of control maxillary incisors at E12.5, whereas overall expression was reduced in mutant embryos (**Figures 6I, J**). At E13.5, LEF1 expression became concentrated within the enamel knot region of control maxillary incisors (**Figure 6K**). This localized enrichment was absent following *Alx1* deletion, accompanied by an overall reduction in LEF1 immunoreactivity (**Figure 6L**). Similar to active β-catenin, LEF1 expression in mandibular incisors remained detectable but was moderately reduced compared with controls (**Figures 6M–P**). Collectively, these findings demonstrate that neural crest-specific deletion of *Alx1* attenuates canonical Wnt signaling during early incisor morphogenesis, with the most pronounced effects observed in maxillary incisors undergoing enamel knot formation.

## 4 Discussion

Tooth morphogenesis depends on precisely coordinated signaling between the dental epithelium and neural crest-derived mesenchyme (Jernvall and Thesleff, 2000; Jussila and Thesleff, 2012; Thesleff, 2003; Tucker and Sharpe, 2004). In the present study, neural crest-specific deletion of *Alx1* did not prevent the initial formation of the incisor tooth germ but severely impaired its subsequent morphogenetic progression. Mutant maxillary incisors initiated normally, as indicated by epithelial thickening and mesenchymal condensation at E12.5, but failed to complete the bud-to-cap transition and did not establish a morphologically or molecularly discernible enamel knot. These findings distinguish *Alx1* from factors required for the initial specification of odontogenic tissues and instead identify it as an important regulator of the signaling interactions that sustain early tooth morphogenesis after initiation.

The enamel knot is a transient epithelial signaling center that coordinates tooth germ growth, epithelial folding, and pattern formation through the localized expression of morphoregulatory signals (Cho et al., 2007; Jernvall et al., 1994; Thesleff and Jernvall, 1997; Thesleff et al., 2001). Its establishment depends on inductive interactions with the underlying dental mesenchyme. Because *Alx1* was deleted specifically in neural crest-derived mesenchyme, the loss of enamel knot-associated epithelial organization and signaling in mutant incisors is consistent with a requirement for *Alx1*-dependent mesenchymal activity in supporting the formation or stabilization of this epithelial signaling center. At E12.5, epithelial *Shh* expression was largely preserved, consistent with the relatively normal initiation of the tooth germ. At E13.5, however, mutant maxillary incisors failed to establish the restricted *Shh*-positive domain characteristic of the enamel knot. This stage-dependent disruption suggests that *Alx1* is not essential for the initial activation of epithelial odontogenic signaling but may become increasingly important as reciprocal epithelial–mesenchymal interactions intensify during the bud-to-cap transition.

The reduction of *Bmp4* and *Msx1* provides a plausible molecular context for this developmental arrest. The *Bmp4-Msx1* regulatory module is a central component of early odontogenic mesenchymal signaling and contributes to the maintenance of epithelial-mesenchymal communication required for progression beyond the bud stage (Jia et al., 2016, 2013). In *Alx1*-deficient maxillary incisors, mesenchymal *Bmp4* and *Msx1* expression was already reduced at E12.5 and became markedly impaired at E13.5, coinciding with the failure of enamel knot formation. However, these expression changes should not be interpreted as evidence that *Alx1* directly or selectively regulates the *Bmp4-Msx1* module. In particular, the E13.5 molecular changes were detected at a stage when mutant tooth germs had already exhibited clear morphological evidence of developmental arrest. Thus, some of the observed changes in *Bmp4* and *Msx1* may represent secondary consequences of altered developmental progression rather than primary effects of *Alx1* loss. Their reduced expression may reflect direct transcriptional control, indirect regulation through other mesenchymal factors, or progressive disruption of tissue interactions as the mutant tooth germ diverges from normal development.

The relative preservation of PAX9 expression is informative in this context. Both MSX1 and PAX9 are key mesenchymal transcription factors required for early tooth development, and loss of either factor can lead to developmental arrest (Peters et al., 1998; Satokata and Maas, 1994). Nevertheless, PAX9 expression remained largely intact following *Alx1* deletion, despite the pronounced reduction in MSX1. This differential response suggests that the molecular consequences of *Alx1* deficiency are not uniform across odontogenic mesenchymal regulators. The maintenance of PAX9 may also help explain why tooth initiation still occurred in mutant embryos, although it was insufficient to support subsequent enamel knot formation and morphogenetic progression.

Canonical Wnt signaling was also attenuated in *Alx1*-deficient incisors. Active β-catenin and LEF1 were reduced in both epithelial and mesenchymal compartments, with the most pronounced loss occurring in the maxillary epithelial region where the enamel knot normally forms. This finding is consistent with the transcriptomic enrichment of Wnt-associated genes and the altered expression of Wnt modulators, including *Wif1* and *Dkk1*. Canonical Wnt signaling functions in both tissue compartments during early odontogenesis and interacts extensively with BMP- and Msx1-dependent regulatory networks (Chen et al., 2009; Fujimori et al., 2010; Jia et al., 2016; Kwon et al., 2017; Liu et al., 2008; O'Connell et al., 2012). Accordingly, the simultaneous reduction of BMP4, MSX1, active β-catenin, and LEF1 is unlikely to represent disruption of a simple linear pathway. A more plausible interpretation is that *Alx1* contributes to the stability of an interconnected odontogenic signaling network in the dental mesenchyme, and that loss of this regulatory support secondarily compromises epithelial Wnt activity and enamel knot establishment. Because Wnt signaling was assessed using pathway-associated markers rather than direct functional manipulation, the present study demonstrates attenuation of canonical Wnt activity but does not establish its precise position relative to *Alx1* or the *Bmp4-Msx1* module.

A notable feature of the phenotype was the substantially greater sensitivity of maxillary incisors to *Alx1* loss. Maxillary incisors underwent complete developmental arrest and subsequent agenesis, whereas mandibular incisors were retained and progressed through morphogenesis, although they were smaller and showed altered dentin deposition. Interestingly, the greater sensitivity of maxillary structures in our model is broadly consistent with the regional pattern described in severe human *ALX1*-related FND, in which developmental disruption prominently involves frontonasal and maxillary processes, whereas mandibular processes have been reported to be relatively spared (Uz et al., 2010). Regional differences between maxillary and mandibular dentitions have been observed following disruption of several developmental regulators, supporting the concept that upper and lower teeth are governed by overlapping but non-identical gene regulatory programs (Faruangsaeng et al., 2022; Jia et al., 2013). The particularly severe maxillary phenotype may reflect differences in the developmental sensitivity of maxillary and mandibular incisors to *Alx1* loss, including differences in their signaling requirements, temporal dynamics, or compensatory mechanisms. The current study does not distinguish among these possibilities. Comparative transcriptional or chromatin-level analyses of maxillary and mandibular dental mesenchyme at stages preceding overt morphological divergence will be required to determine why mandibular incisors retain greater developmental competence in the absence of *Alx1*.

The dynamic expression of *Alx1* further suggests that its function may extend beyond early morphogenesis. During the early stages examined, *Alx1* was broadly expressed in the dental mesenchyme, consistent with a role in regulating mesenchymal competence and epithelial-mesenchymal signaling. At E16.5, its expression became enriched in differentiating odontoblasts. Together with the altered dentin matrix observed in mutant mandibular incisors, this later expression pattern raises the possibility that *Alx1* also contributes to odontoblast maturation or dentin matrix production. This stage-dependent shift in expression is consistent with the behavior of other homeobox transcription factors that participate in both early patterning and later differentiation processes (Carpinelli et al., 2017; Schock and LaBonne, 2020; Yan et al., 2020). However, the present study was primarily designed to investigate early incisor morphogenesis, and the severe developmental arrest of maxillary incisors precluded direct assessment of later differentiation in that region. Additional temporally controlled deletion models will be necessary to separate the early morphogenetic and later odontoblast-associated functions of *Alx1*.

The findings also have potential relevance to *ALX1*-associated frontonasal dysplasia type 3. Biallelic pathogenic variants in *ALX1* cause a spectrum of severe frontonasal and midfacial abnormalities, including facial and palatal clefting and defects involving maxillary developmental structures (Pini et al., 2020; Uz et al., 2010), whereas protruding teeth have been reported in a milder *ALX1*-related phenotype (Ullah et al., 2017). However, detailed dental phenotyping remains limited, and specific abnormalities of tooth morphogenesis have not been systematically characterized in affected individuals. Our mouse model demonstrates that loss of *Alx1* in neural crest-derived tissues can directly disrupt incisor development and identifies failure of enamel knot formation as a possible developmental basis for such dental defects. Nevertheless, caution is required when extrapolating from mouse incisors to the human dentition. The continuously growing mouse incisor has distinct developmental and anatomical characteristics, and the dental phenotypes of individuals with FND3 have not yet been sufficiently characterized to establish direct correspondence with the mouse phenotype. The present findings therefore provide a developmental framework for understanding how *ALX1* deficiency may affect odontogenesis rather than a complete model of the human dental manifestations.

In conclusion, this study identifies *Alx1* as an essential regulator of enamel knot formation and incisor morphogenesis in neural crest-derived dental mesenchyme. Loss of *Alx1* is associated with perturbation of the *Bmp4-Msx1*-related odontogenic program and attenuation of canonical Wnt signaling, together with failure of enamel knot formation and markedly greater developmental impairment of maxillary than mandibular incisors. Although the causal hierarchy among these molecular alterations remains unresolved, the findings establish a previously unrecognized developmental role for *Alx1* in tooth morphogenesis and highlight the region-specific sensitivity of the maxillary dentition to *Alx1* deficiency.

## Data Availability

The datasets presented in this study can be found in online repositories. The names of the repository/repositories and accession number(s) can be found below: https://www.ncbi.nlm.nih.gov/geo/, GSE336212.
