# Supplementary material for: Alx1 is required for enamel knot formation during incisor morphogenesis
Source: Front Physiol. 2026 Sep 3;17:1939236. doi: 10.3389/fphys.2026.1939236 (PMC13581496; doi:10.3389/fphys.2026.1939236)
Supplement: Supplementary file 1 [file DataSheet1.pdf]

Supplementary Material

Supplementary Figures

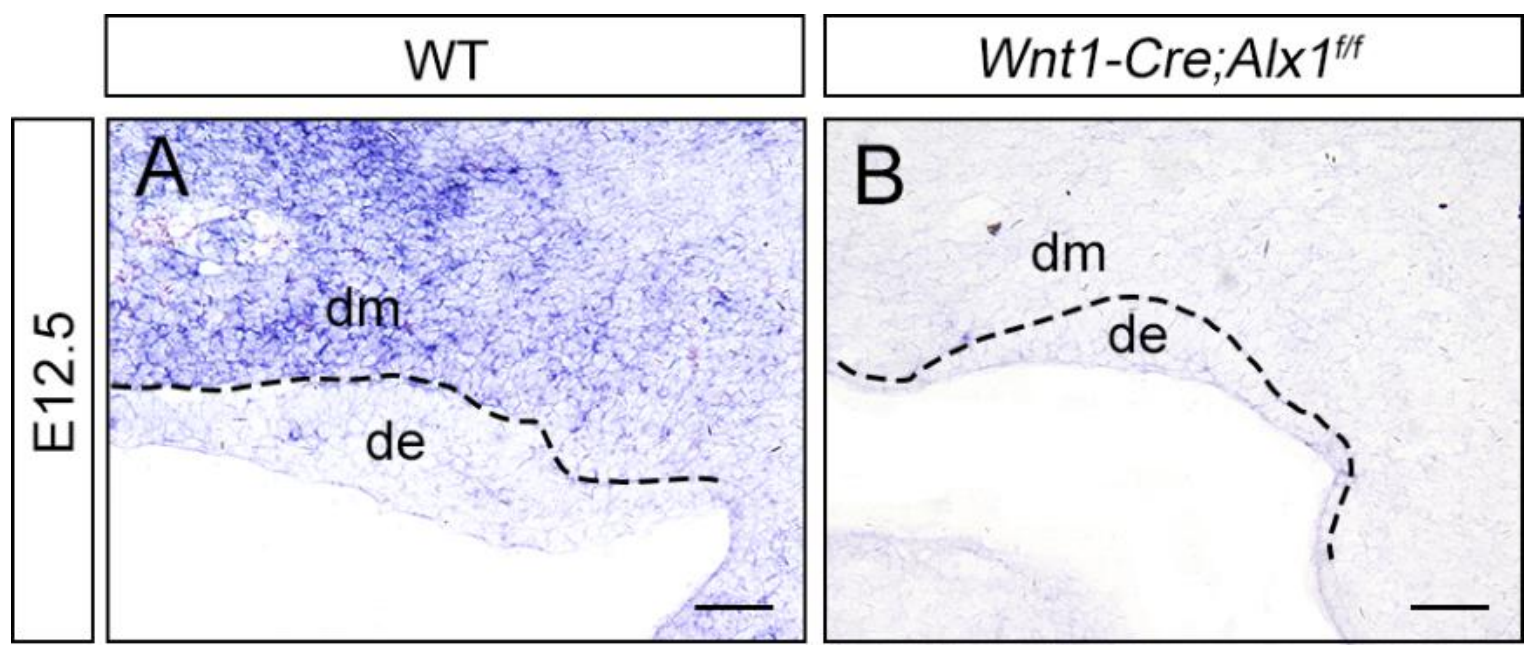

Supplementary Figure 1. Efficient deletion of *Alx1* in the dental mesenchyme of *Wnt1-Cre;Alx1$^{f/f}$* embryos. In situ hybridization analysis of *Alx1* expression in the maxillary incisor region at E12.5. Robust *Alx1* expression is detected in the dental mesenchyme of WT embryos (A), whereas expression is markedly reduced in *Wnt1-Cre;Alx1$^{f/f}$* embryos (B), confirming efficient neural crest-specific deletion of *Alx1*. Dashed lines outline the dental epithelium. dm, dental mesenchyme; de, dental epithelium. Scale bars, 100 µm.

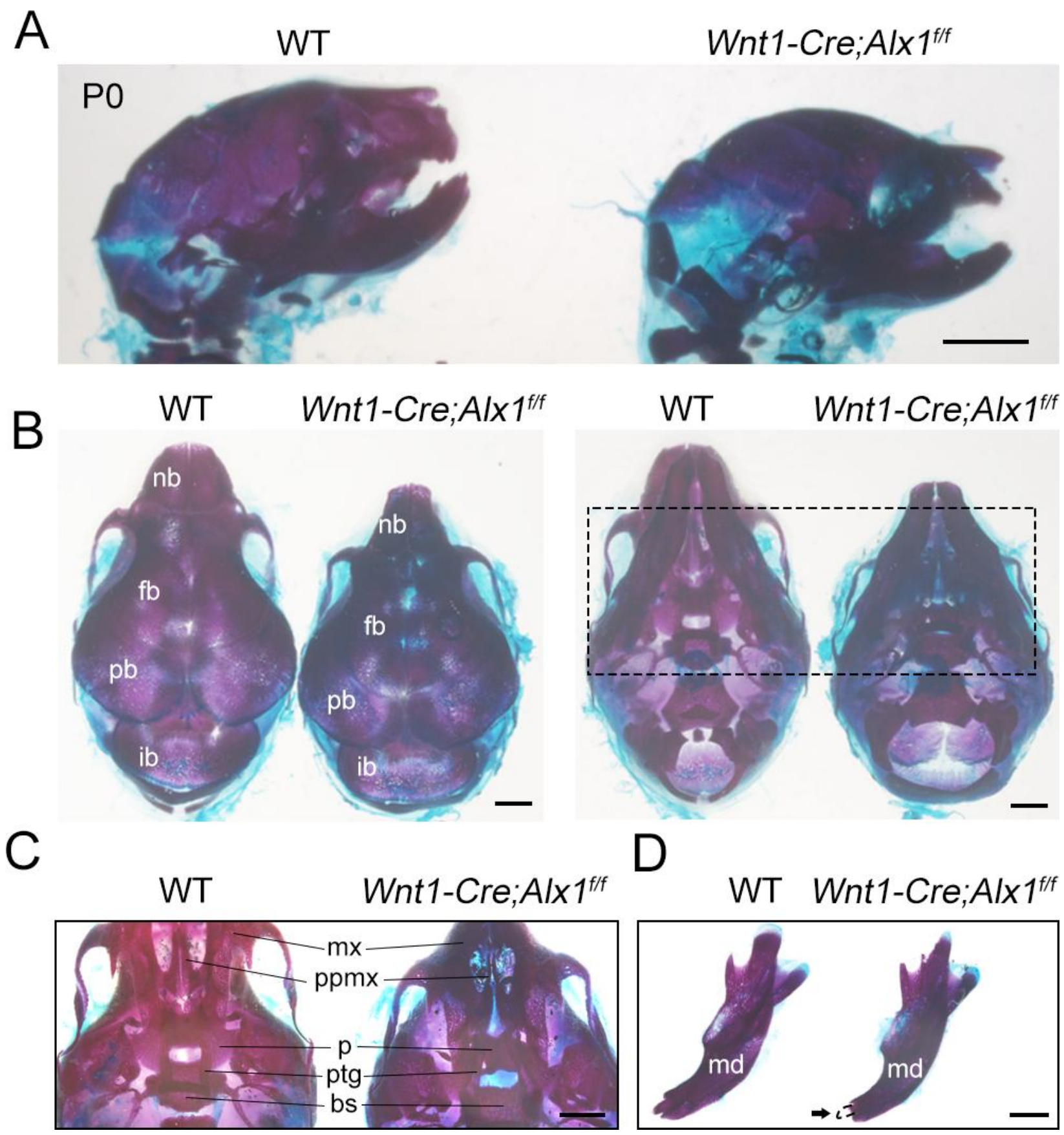

Supplementary Figure 2. Craniofacial skeletal abnormalities in *Wnt1-Cre;Alx1$^{f/f}$* mice. (A) Lateral views of the craniofacial skeletons of P0 WT and *Wnt1-Cre;Alx1$^{f/f}$* mice showing the altered craniofacial morphology in mutant mice. Scale bars, 1 mm. (B) Dorsal and ventral views of the skull showing morphological alterations in the anterior craniofacial skeletal region, including the nasal, frontal, and maxillary regions, in mutant mice. (C) Higher-magnification views of the ventral craniofacial skeleton corresponding to the boxed regions in (B), highlighting abnormalities involving the maxillary regions and alterations in the palatal/pterygoid skeletal structures. (D) Lateral views of the mandible showing reduced length of the mandibular incisors in mutant mice compared with controls (arrows). nb, nasal bone; fb, frontal bone; pb, parietal bone; ib, interparietal bone; mx, maxilla; ppmx, palatal process of maxilla; p, palatine; ptg, pterygoid; bs, basisphenoid; md, mandible. Scale bars, 500 µm (B-D).
